# CTBP1 and metabolic syndrome induce an mRNA and miRNA expression profile critical for breast cancer progression and metastasis

**DOI:** 10.18632/oncotarget.24486

**Published:** 2018-02-13

**Authors:** Paula L. Farré, Georgina D. Scalise, Rocío B. Duca, Guillermo N. Dalton, Cintia Massillo, Juliana Porretti, Karen Graña, Kevin Gardner, Paola De Luca, Adriana De Siervi

**Affiliations:** ^1^ Laboratorio de Oncología Molecular y Nuevos Blancos Terapéuticos, Instituto de Biología y Medicina Experimental, CONICET, Buenos Aires, Argentina; ^2^ Department of Pathology and Cell Biology, Columbia University Medical Center, New York, New York, USA

**Keywords:** CTBP1, miRNAs, metabolic syndrome, breast cancer, metastasis

## Abstract

Metastatic breast cancer (BrCa) is still one of the main causes of cancer death in women. Metabolic syndrome (MeS), a risk factor for BrCa, is associated to high grade tumors, increased metastasis and recurrence of this disease. C-terminal binding protein 1 (CTBP1) is a co-repressor of tumor suppressor genes that is activated by low NAD^+^/NADH ratio. Previously, we demonstrated that CTBP1 hyperactivation by MeS increased tumor growth in MDA-MB-231-derived xenografts regulating several genes and miRNAs. In this work, our aim was to elucidate the role of CTBP1 and MeS in BrCa metastasis. We found that CTBP1 protein diminished adhesion while increased migration of triple negative BrCa cells. CTBP1 and MeS modulated the expression of multiple genes (ITGB4, ITGB6, PRSS2, COL17A1 and FABP4) and miRNAs (miR-378a-3p, miR-146a-5p, let-7e-3p, miR-381-5p, miR-194-5p, miR-494-3p) involved in BrCa progression of MDA-MB-231-derived xenografts. Furthermore, we demonstrated that MeS increased lung micrometastasis and liver neoplastic disease in mice. CTBP1 hyperactivation seems to be critical for MeS effect on BrCa metastasis since CTBP1 depletion completely impaired the detection of circulating tumor cells. Our results highlight CTBP1 and MeS impact on BrCa progression positioning them as key properties to be considered for BrCa patient prognosis and management.

## INTRODUCTION

Breast cancer (BrCa) is the most common type of cancer in women (aside from skin cancer), and it is the main cause of death between women [1]. Even though cancer is influenced by genetic conditions, there are several risk factors such as diet, overweight and sedentary lifestyle that could be determinant in the development of this disease [2].

According to the National Cholesterol Education Program Adult Treatment Panel III (NCEP ATP III) criteria, metabolic syndrome (MeS) is a cluster of pathophysiological disorders including three or more of the following factors: blood-pressure ≥130/85 mm Hg, triglycerides ≥150 mg/dL, abdominal obesity (waist circumference ≥35 inches in women), high density lipoprotein (HDL-C) <50 mg/dL for women and fasting glucose ≥ 110 mg/dL [3, 4]. Recently, a meta-analysis study had shown that MeS is a risk factor for BrCa in general population [5] having a stronger association in post-menopausal women [6]. Beside this association, a retrospective study demonstrated that MeS has a major prevalence in triple negative BrCa (TNBC) rather than in other types [7]. Additionally, MeS is associated to high grade tumors and higher recurrence rate and metastasis of BrCa [8].

Previously, we reported C-terminal binding protein 1 (CTBP1), a transcriptional co-repressor of tumor suppressor genes [9], as a molecular link between MeS and BrCa [10] or prostate cancer [11]. CTBP1 activation occurs after dimerization produced by NAD^+^ or NADH binding. CTBP1 has been proposed as a metabolic cellular sensor since it shows a higher affinity for NADH (>100-fold) compared to NAD^+^ [12]. Furthermore, we found that MeS increased postnatal development of mice mammary gland observed as an induction of terminal bulbs number. Moreover, we detected epithelial changes in mammary ducts with high expression of CTBP1 and CCND1 in MeS mice [10]. CTBP1 and MeS increased breast tumor growth regulating several genes and miRNAs involved in cell proliferation, self-renewal, mammary differentiation, cell communication, metabolic processes and epithelial-to-mesenchymal transition (EMT) in orthotopic xenografts [10].

Metastasis is still the main cause of death for BrCa patients, and around 30% of women with BrCa diagnosed at early stages will progress to metastatic stage. The aim of this work was to investigate the role of CTBP1 and MeS in BrCa metastasis using a MeS experimental model. We found that CTBP1 decreased BrCa cell adhesion and migration through expression modulation of multiple genes and miRNAs involved in BrCa progression. Moreover, CTBP1 and MeS increased circulating tumor cells (CTCs) and metastasis in nude mice.

## RESULTS

### CTBP1 protein regulates cell adhesion and migration of BrCa cells

**Figure 1:**
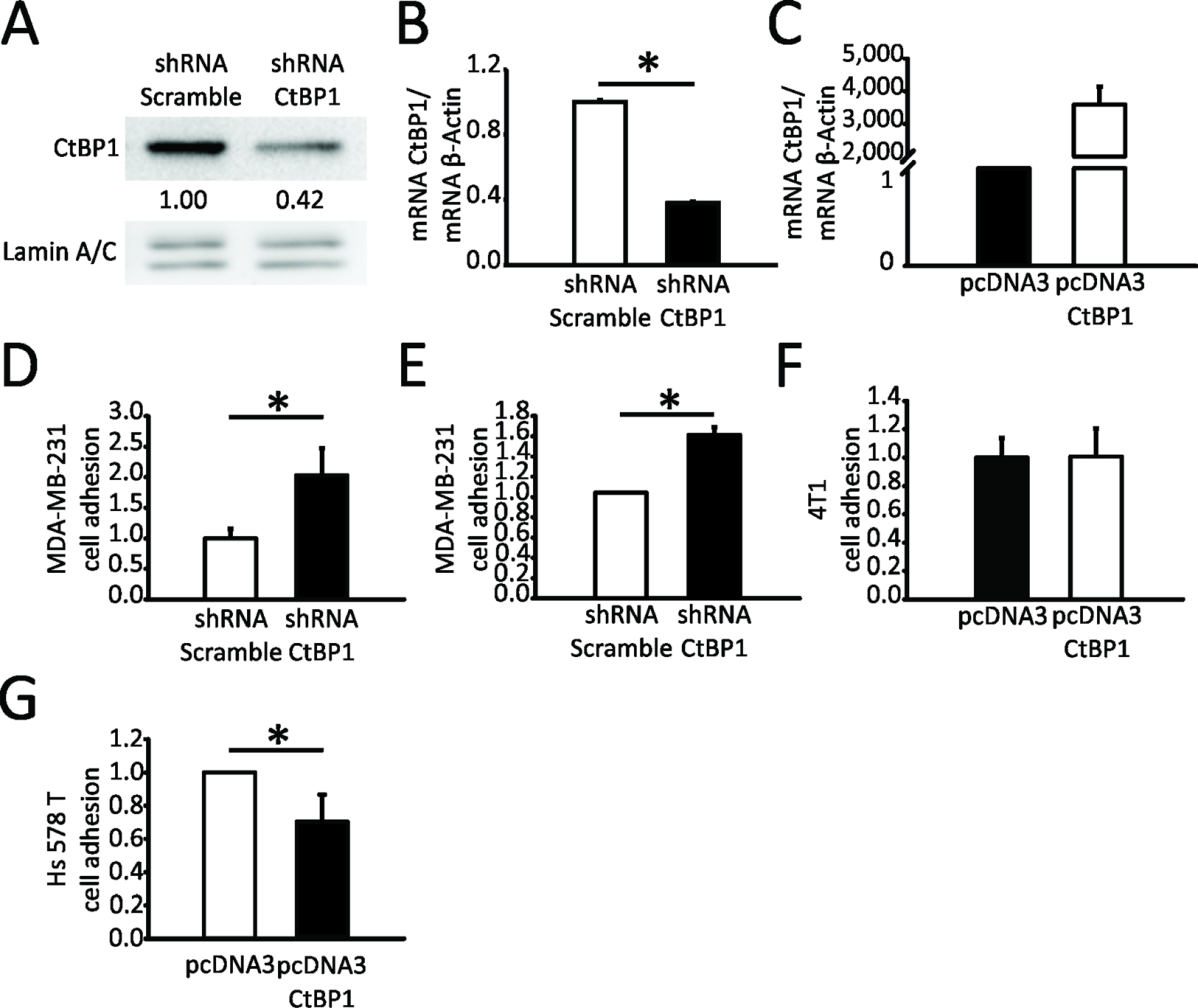
CTBP1 diminishes MDA-MB-231 and Hs578T cell adhesion CTBP1 expression was determined in MDA-MB-231 CTBP1 stable depleted (shRNA CTBP1) or control (shRNA Scramble) cells by (**A**) WB and (**B**) RT-qPCR. WB quantification of each band using Image J software is shown. Data were normalized to LMNA and control cells. CTBP1 mRNA expression levels were normalized to ACTB and control (^*^p value < 0.05). (**C**) CTBP1 mRNA levels were determined in 4T1 cells transiently transfected with CTBP1 overexpression (pcDNA3 CTBP1) or control (pcDNA3) vectors by RT-qPCR and normalized to ACTB and control. Cell adhesion assay was performed in MDA-MB-231 shRNA CTBP1 or shRNA Scramble cells without (**D**) or with (**E**) collagen matrix. The mean and SD of one representative experiment (*n* = 2) with triplicates is shown. Data were normalized to protein and control (^*^p value < 0.05). (**F**) Cell adhesion assay was performed in 4T1 pcDNA3 CTBP1 or pcDNA3 cells. The mean and SD of one representative experiment (*n* = 2) with triplicates is shown. Data were normalized to control. (**G**) Cell adhesion assay was performed in Hs578T pcDNA3 CTBP1 or pcDNA3 cells. The mean and SD of one representative experiment (*n* = 2) with triplicates is shown. Data were normalized to control.

To analyze CTBP1 role in breast tumor progression, we first analyzed CTBP1 modulation effect on MDA-MB-231, 4T1 and Hs578T BrCa cell adhesion and migration, both cell lines representing advanced stages of TNBC. *In vitro* cell adhesion assay with or without collagen matrix was performed using stable transfected MDA-MB-231 cells with diminished (shRNA CTBP1) or control (shRNA Scramble) expression of *CTBP1*. CTBP1 protein and mRNA levels in the transfected cell lines were assessed by WB (Figure 1A) and RT-qPCR (Figure 1B–1C), respectively. CTBP1 depletion increased MDA-MB-231 cell adhesion, both in the presence or absence of collagen matrix (Figure 1D, 1E). Additionally, 4T1 and Hs578T cells were transiently transfected with CTBP1 plasmid (pcDNA3 CTBP1) or control (pcDNA3) and cell adhesion was determined. As shown in Figure 1F and 1G, CTBP1 significantly decreased Hs578T cell adhesion without changes in 4T1 cells.

**Figure 2:**
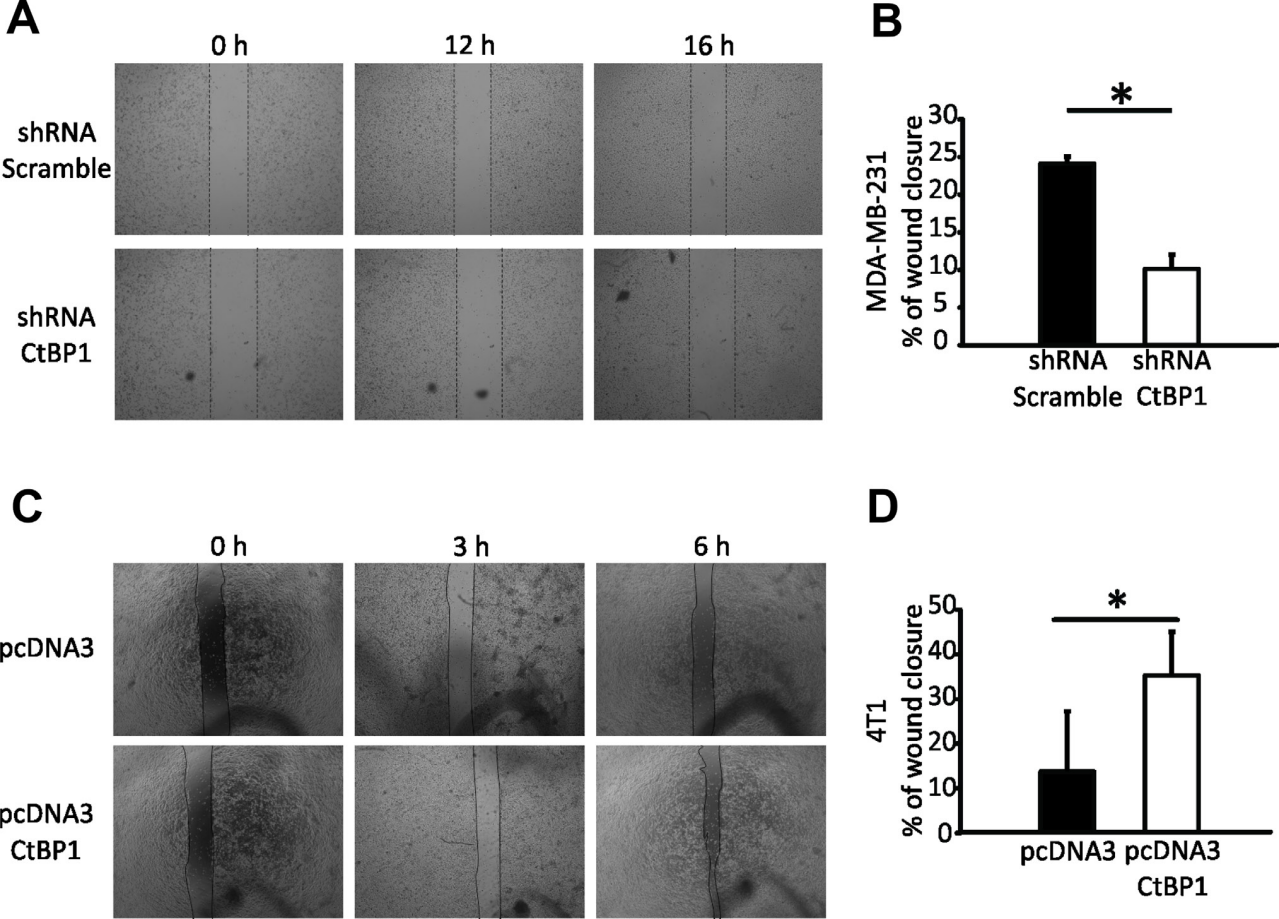
CTBP1 modulates BrCa cell migration (**A**) Wound healing assay was performed using MDA-MB-231 CTBP1 stable depleted (shRNA CtBP1) or control (shRNA Scramble) cells. Representative pictures of wound at 0, 12 and 16 h from 2 independent experiments with triplicates are shown. (**B**) Percentage of wound closure of MDA-MB-231 shRNA CTBP1 or shRNA Scramble cells is shown as mean and SD of one representative experiment (*n* = 2) with triplicates (^*^p value < 0.05). (**C**) Wound healing assay was performed using 4T1 pcDNA3 CTBP1 or pcDNA3. Representative pictures of wound at indicated times from 2 independent experiments with triplicates are shown. (**D**) Percentage of wound closure of 4T1 pcDNA3 CTBP1 or pcDNA3 cells is shown as mean and SD of one representative experiment (*n* = 2) with triplicates (^*^p value < 0.05).

Cell migration of MDA-MB-231 CTBP1 depleted cells (Figure 1A) and 4T1 CTBP1 overexpressing cells (Figure 1C) were determined by wound healing assay. We found that CTBP1 depletion decreased wound closure of these cell lines, and in turn, CTBP1 overexpression induced migration (Figure 2A–2D).

In summary, CTBP1 diminished cell adhesion and increased cell migration, both initial processes for tumor progression, in TNBC cells.

### CTBP1 and MeS modulate multiple genes and miRNAs involved in BrCa progression

**Figure 3:**
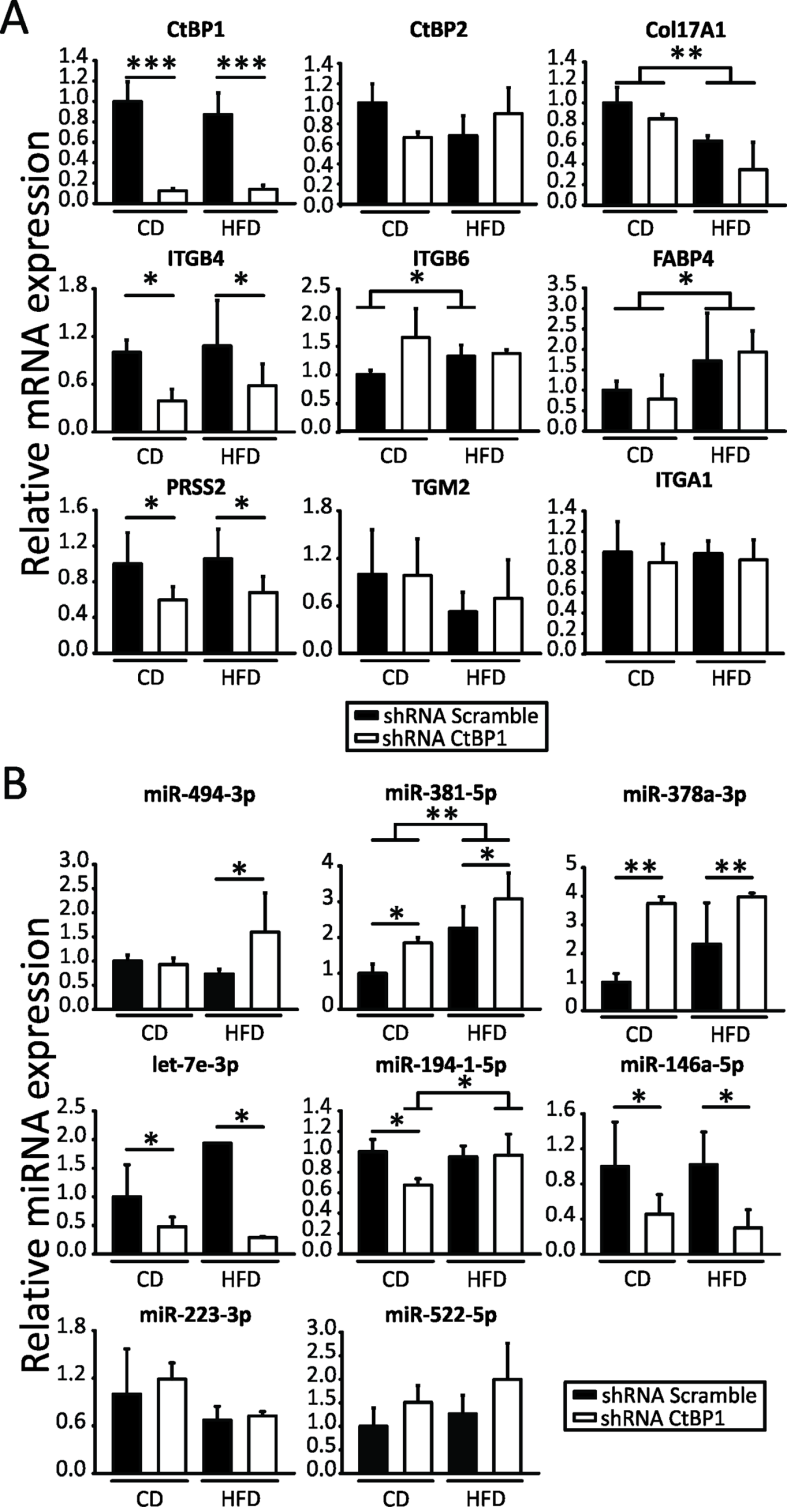
CTBP1 and MeS modulate multiple genes and miRNAs involved in BrCa progression Expression of the indicated mRNAs (**A**) and miRNAs (**B**) in xenografts from CD or HFD mice inoculated with MDA-MB-231 shRNA CTBP1 or shRNA Scramble cells were determined by RT-qPCR using specific primers. Data were normalized to ACTB and control for mRNAs or to geometric mean of miR-103a-3p, miR-191-5p and miR-17-5p and control tumors for miRNAs (^*^p value < 0.05; ^**^p value < 0.01; ^***^p value < 0.001).

To study the relevance of CTBP1 and MeS in BrCa progression, female *nu*/*nu* mice were chronically fed with control diet (CD) or high fat diet (HFD) and inoculated in the mammary fat pad (MFP) with CTBP1-depleted (shRNA CTBP1) or control (shRNA Scramble) MDA-MB-231 cells. Xenograft samples were collected for total RNA isolation and expression of genes involved in key processes for BrCa progression was determined by RT-qPCR. First, mRNA levels of CTBP family members were assessed in order to check that *CTBP1* expression was diminished without changes in *CTBP2* during the experiment (Figure 3A).

Then, we assessed expression of cell adhesion genes: *COL17A1, FABP4, ITGA1, ITGB4, ITGB6, TGM2*; cell migration genes: *COL17A1, FABP4, ITGB4, ITGB6, PRSS2, TGM2* and cell invasion genes: *FABP4, ITGB4, ITGB6, PRSS2, TGM2*. We found that CTBP1 significantly modulated *ITGB4* and *PRSS2* genes while MeS regulated *COL17A1* and *FABP4* expression (Figure 3A). Interestingly, we found that *ITGB6* regulation by MeS occurred only in xenografts with high CTBP1 expression (Figure 3A).

Previously, we identified 42 miRNAs involved in metabolism, cell cycle and cell communication, regulated by CTBP1 in BrCa orthotopic xenografts generated in MeS mice [10]. In this work, to elucidate which of these miRNAs could be crucial for BrCa development and tumor progression, we performed a reactome analysis using the bioinformatic resource miRSystem based on the number of biological processes regulated by each miRNA. We identified a cluster of miRNAs with relevant roles in cell proliferation (miR-378a-3p, miR-146a-5p and miR-381) and tumor progression (miR-378a-3p, miR-146a-5p, miR-381, miR-223-3p, miR-494-3p, miR-940, miR-433, miR-522 and miR-637) (Supplementary Table 1). Based on this analysis we validated these miRNAs by RT-qPCR. Also, considering their functions in BrCa progression, we included for further studies let-7e-3p [13] and miR-194-1-5p [14], both identified as CTBP1-regulated miRNAs in the microarray. As shown in Figure 3B, using miRNA RT-qPCR we found that CTBP1 modulated miR-494-3p, miR-381-5p, miR-378a-3p, let-7e-3p, miR-194-1-5p and miR-146a-5p in MDA-MB-231-derived xenografts (Figure 3B). Furthermore, MeS induced miR-381-5p and miR-194-1-5p expression (Figure 3B). We were not able to validate the expression of miR-940, miR-433-3p and miR-637 by RT-qPCR stem loop method.

Altogether these results suggest that CTBP1 and MeS regulate BrCa progression throughout modulation of multiple genes and miRNAs.

### MeS and CTBP1 increase BrCa lung metastases and liver neoplastic disease

**Figure 4:**
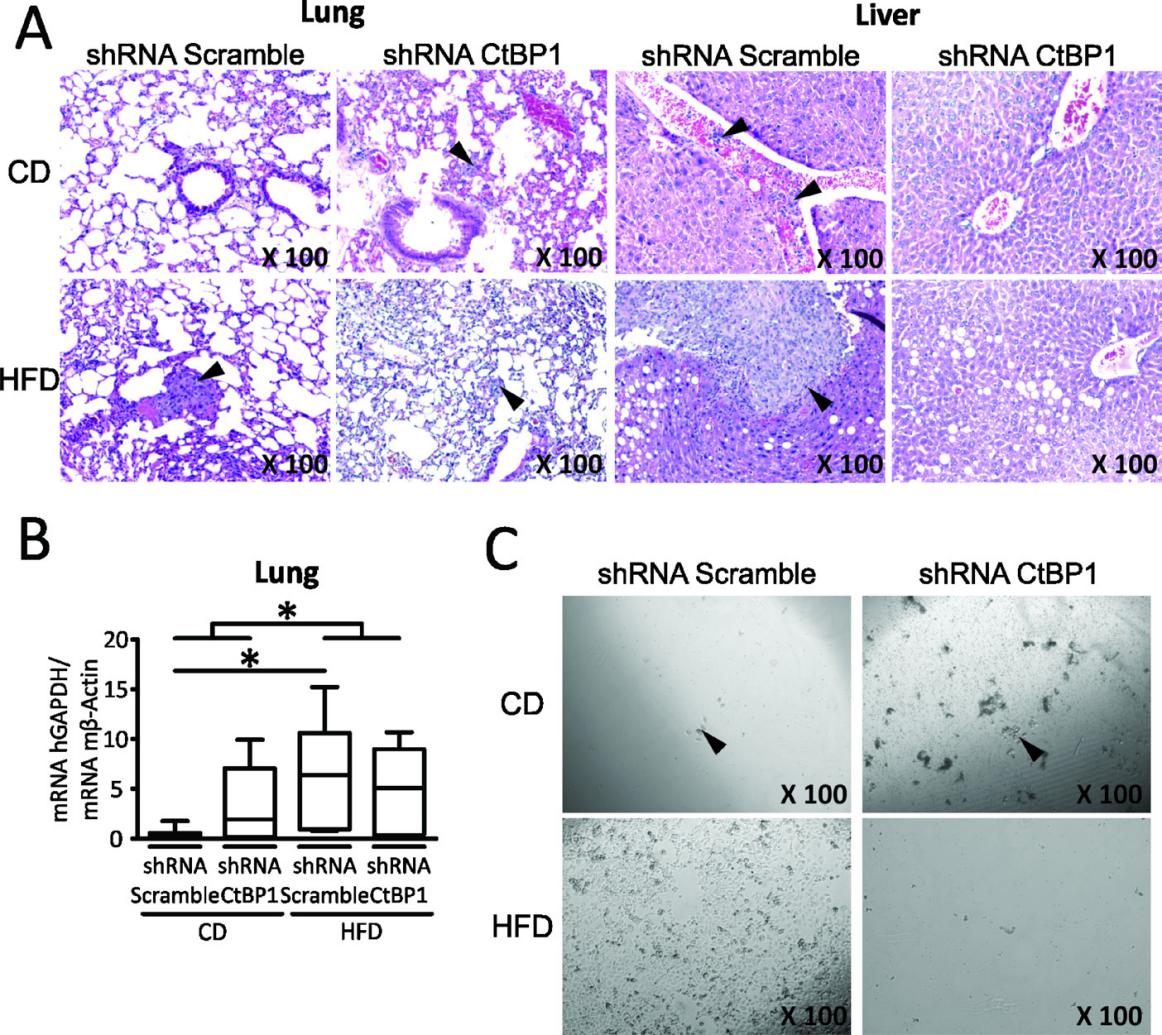
MeS and CTBP1 increase CTCs inducing BrCa lung metastasis and liver neoplastic disease Mice were fed with CD or HFD and inoculated with MDA-MB-231 shRNA CTBP1 or shRNA Scramble cells. Mice were sacrificed 45 days post-injection and soft tissue and blood samples were collected. (**A**) Lung and liver H&E staining is shown. Arrowheads indicate micrometastasis. Magnifications ×100. (**B**) Presence of human BrCa cells in lung of mice was analyzed by determining human GAPDH expression by RT-qPCR using specific human primers. Data were normalized to mouse ACTB (^*^p value < 0.05). (**C**) Clonogenic assay for detection of CTCs present in blood of mice was performed in medium supplemented with FBS and puromycin (1 μg/mL). Representative pictures are shown (magnifications ×100). Arrowheads indicate colonies.

We next analyzed the effect of CTBP1 and MeS on BrCa metastasis. Thus, MeS female *nu/nu* mice or control were inoculated in the MFP with MDA-MB-231 shRNA CTBP1 or shRNA Scramble cells. After 45 days post-injection, lung and liver samples were collected for macroscopic, histological and RT-qPCR analysis. As previously reported for MDA-MB-231 cell line, macroscopic analysis did not reveal the presence of metastasis in all experimental groups; however, hematoxylin and eosin (H&E) staining and histological analysis allowed detection of micrometastases in both lung and liver. We found that hyperactivation of CTBP1 by MeS increased the percentage of mice with lung metastases and liver neoplastic disease, which includes hepatic micometastasis and intravascular tumor cells (Figure 4A and Table 1). Moreover, MeS mice inoculated with control cells developed micrometastasis bigger than other groups, suggesting that CTBP1 hyperactivation by MeS increase both development of metastasis and aggressiveness of secondary tumors (Figure 4A). We also detected human BrCa cells in lungs by RT-qPCR using specific human GAPDH primers. MeS increased the amount of human BrCa cells in lungs, and this effect was enlarged in mice injected with higher CTBP1 expression cells (shRNA Scramble) (Figure 4B). Also, CTCs in mice blood were identified by clonogenic assays (Figure 4C and Table 2). In agreement to these results, we found that CTBP1-depletion completely impaired CTCs from MeS mice.

**Table 1:** Quantification of lung metastasis and liver neoplastic disease

Experimental group	Lung metastasis (% of mice)	Liver neoplastic disease (% of mice)
shRNA Scramble CD (*n* = 7)	0	14 iv
shRNA CTBP1 CD (*n* = 9)	11	0
shRNA Scramble HFD (*n* = 6)	33	33 iv
shRNA CTBP1 HFD (*n* = 7)	14	0

Mice were chronically fed with HFD or CD and inoculated with MDA-MB-231 shRNA CTBP1 or shRNA Scramble. Forty-five days post-injection mice were sacrificed and presence of micrometastasis to lung and liver was determined by histological analysis. Percentage of mice with lung metastasis or liver neoplastic disease of each experimental group is informed. In: intravenous.

**Table 2:** Quantification of CTCs from mice

Experimental group	% of mice with CTCs
shRNA Scramble CD (*n* = 6)	33
shRNA CTBP1 CD (*n* = 6)	50
shRNA Scramble HFD (*n* = 6)	33
shRNA CTBP1 HFD (*n* = 6)	0

Mice were chronically fed with HFD or CD and inoculated with MDA-MB-231 shRNA CTBP1 or shRNA Scramble. Forty four days post-injection mice were sacrificed and presence of CTCs in blood samples were assessed. Percentage of mice with CTCs of each experimental group is shown.

Altogether, these results suggest that CTBP1 and MeS are key activators of BrCa progression and metastasis.

## DISCUSSION

Even though BrCa mortality has been reduced over the last decade [15], metastatic BrCa is still one of the main causes of cancer death in women, being the improvement in the survival rates of patients with metastatic BrCa a major concern for public health [16]. Once metastasis occurs, BrCa becomes a systemic disease and the 5-year survival rate decreases to 20% [17]. As well reviewed by *Lim et al.*, the heterogeneity between patients will determine the course of the disease and it is crucial for treatment decisions [16]. Several efforts are being made to elucidate the main sources of this heterogeneity. In addition, we propose that patient health conditions that impact on cancer progression and morbidity, together with their associated molecular targets, should be considered for treatment decisions and therapies development. Based on this, MeS constitutes a health condition related to BrCa recurrence and metastasis, that might be identified for differential patient management [8]. To understand the molecular mechanism underlying BrCa progression connected to MeS might be a key aspect for these patients. Previously, we identified CTBP1 hyperactivation by MeS as an inductor of breast carcinogenesis and tumor growth in mice. In this work, we advocated to the study CTBP1 and MeS role over BrCa metastasis.

*In vitro* approaches allowed us to elucidate CTBP1 protein effect on adhesion and migration of TNBC cells. We first demonstrated that CTBP1 protein diminished MDA-MB-231 and Hs578T cell adhesion. Several studies have shown that CTBP1 modulates the expression of numerous EMT markers, such as E-cadherin, in BrCa cells [9, 10]. *Di et al.* and *Deng et al.* demonstrated that CTBP1 induces a mesenchymal phenotype, which is associated with low adhesion, high migration and invasion capabilities through the regulation of multiple genes [18, 19]. However, this is the first study that demonstrates cell adhesion regulation by CTBP1 in BrCa cells. We also found that CTBP1 protein increased the migratory capability of TNBC MDA-MB-231 and 4T1 cells. These results support previous studies showing that CTBP1 increases migration of hormone sensitive MCF-7 BrCa cells [18] and other cell types such as glioma cancer cells [20].

Previously, we generated a MeS-like experimental model and found that CTBP1 and MeS increased breast tumor growth by regulating multiple genes and miRNAs involved in cell proliferation, progenitor cells phenotype, EMT, mammary development and cell communication [10]. To elucidate CTBP1 and MeS effect on BrCa progression *in vivo*, here we analyzed expression levels of genes involved in key steps of tumor progression in xenografts generated in the MeS-like model. Moreover, we showed that CTBP1 induced *ITGB4* and *PRSS2*, while MeS regulated *COL17A1* and *FABP4* genes. In addition, MeS induced *ITGB6* only in CTBP1-high expression xenografts, suggesting that CTBP1 activation by MeS could be critical for *ITGB6* expression. These results support our previous studies showing that, *in vivo*, CTBP1 and MeS regulate the mesenchymal markers Vimentin and Slug [10]. Importantly, integrins dysregulation is associated with cancer development and progression, since these are heterodimeric cell surface receptors critical for adhesion, migration, invasion, growth, survival and differentiation [21]. Interestingly, ITGB4 constitutes a marker of basal-like tumors [22] and it is a downstream effector of cell migration mediated by the mesenchymal marker Vimentin [23], which promotes BrCa invasion through the regulation of cytoskeleton dynamics [24]. Thus, CTBP1 emerges as a master regulator of EMT in BrCa inducing Vimentin and its downstream target ITGB4. Also, ITGB6 is a known inductor of cell invasion and is a marker of poor prognosis in several cancer types, including BrCa [25–28]. Altogether, these results suggest that CTBP1 hyperactivation by MeS is a critical event that might be considered for prognosis assessment in BrCa patients.

CTBP family proteins are encoded by 2 paralogous genes, CTBP1 and CTBP2. These proteins display redundant functional and structural similarities, although each of them also has many distinct roles. However, both are oncogenic transcriptional co-regulators overexpressed in many cancer types. In this work we found that only CTBP1 gene expression was modified in the tumors without CTBP2 changes which supports the idea that the findings are attributed only to CTBP1.

Previously, we identified 42 miRNAs regulated by CTBP1 from xenografts generated in MeS mice [10]. Here, we selected a cluster of miRNAs from the microarray data based on their function in tumor growth and progression for further validation by miRNA RT-qPCR. We demonstrated that CTBP1 represses miR-378a-3p and miR-494-3p and induces miR-146a-5p and let-7e-3p expression in MDA-MB-231 xenografts. Furthermore, CTBP1 and MeS regulate miR-381-5p and miR-194-1-5p expression. These results are consistent with previous studies demonstrating that miR-378a-3p expression is reduced in tumor tissue from BrCa patients and its expression is associated with better prognosis in BrCa patients treated with hormone therapy [29]. Also, miR-381-5p expression is reduced in BrCa compared to adjacent tissue [30]. Moreover, miR-381-5p represses cell proliferation, EMT, invasion and migration of MDA-MB-231 [30], suggesting that one of the mechanisms involved in CTBP1 role in BrCa adhesion and migration is miR-381-5p repression. Interestingly, miR-194-1-5p, induced by both CTBP1 and MeS, is increased in serum from BrCa patients with recurrence compared to patients without recurrence [14]. However, other studies determined that miR-146a-5p and let-7e-3p expression, induced by CTBP1 in MDA-MB-231 xenografts, correlates with better survival in BrCa patients [13, 31]. Furthermore, miR-494-3p repressed by CTBP1 hyperactivation by MeS is increased in patients with metastatic BrCa compared to patients without metastasis [32].

Finally, this is the first report describing CTBP1 role in BrCa metastasis, and more important how this process can be influenced by MeS. We found that MeS and CTBP1 increased lung micrometastasis and liver neoplastic disease in mice. Importantly, we found that CTBP1 expression is crucial for BrCa cells to reach the blood stream, since we observed that CTCs, one of the major steps for metastatic cascade, were dramatically decreased by CTBP1-depletion.

In summary, in this work we developed an experimental model that resembles MeS effect on BrCa metastasis. Moreover, we elucidated a molecular mechanism that explains MeS association with BrCa metastasis based on metastatic cascade activation by CTBP1 throughout the regulation of multiple EMT-related genes and miRNAs.

## MATERIALS AND METHODS

### Cell culture and transfection

MDA-MB-231 and Hs578T cells and its derivatives were grown with DMEM medium (GIBCO) supplemented with 10% of fetal bovine serum (FBS) and antibiotics. Insulin was added to Hs578T cells. 4T1 cells were cultivated with RPMI 1640 medium (Invitrogen) supplemented with 10% of FBS and antibiotics. Hs578T pcDNA3 CTBP1 and pcDNA3 cells and 4T1 pcDNA3 CTBP1 and pcDNA3 cells were generated by transient transfection as previously described [10]. MDA-MB-231 shRNA Scramble and MDA-MB-231 shRNA CTBP1 stable cell lines were previously generated by lentiviral transduction [10]. Stable transfected cells were selected with 2 µg/mL puromycin (Sigma-Aldrich) for 7 days and then maintained with puromycin (1 µg/mL).

### Western blot analysis (WB)

MDA-MB-231 cells were lysed and immunoblotted as previously described [33] using specific antibodies against CTBP1 (BD Transduction Laboratories) and LMNA (Santa Cruz Biotechnology Inc.) proteins. Protein quantification was performed using Image J 1.48 software.

### RNA isolation, cDNA synthesis and qPCR (RT-qPCR)

Total RNA isolation was performed using Tri Reagent (Genbiotech, Buenos Aires, Argentina). cDNA was synthesized from 2 μg of RNA using M-MLV Reverse Transcriptase (Promega). qPCR was performed as previously described [11] using Taq polymerase (Embiotec, Buenos Aires, Argentina) in a StepOne Plus Real Time PCR (Applied Biosystems). Data were normalized to ACTB and control. Primer sequences used are shown in Supplementary Table 2.

### miRNA retrotranscription and qPCR (miRNA RT-qPCR)

miRNAs were retrotranscribed using stem-loop method as previously described [34] with some modifications. Briefly, 100 ng of total RNA were preheated in 14 μL containing 0.07 μM of stem-loop primer at 70°C during 5 min. Then, retrotranscription was performed using M-MLV Reverse Transcriptase (Promega) and incubated in MyGenie96 Thermal Block (Bioneer) for 30 min at 16°C, 50 min at 37°C and 15 min at 70°C. qPCR was performed in 25 μL with 0.05-1 μL RT product, 1U Taq DNA polymerase (Pegasus), 4 mM MgCl_2_, 0.2mM dNTPs, 3 × 10^-5^ μL Sybrgreen (Sigma), 0.1 μM forward primer and 0.1 μM reverse primer. The reactions were incubated in StepOne Plus Real Time PCR (Applied Biosystems) at 94°C for 2 min, followed by 40 cycles of 95°C for 15 s, annealing temperature for 20 s and 72°C for 25 s. All reactions were run in duplicate. The expression levels of miRNAs were normalized to the geometric mean of miR-103a-3p, miR-191-5p and miR-17a-5p levels as previously described [35]. Primer sequences for miRNA RT-qPCR are listed in Supplementary Table 2.

### Cell adhesion assay

Cell adhesion assay was performed using 96-well tissue culture plates with or without collagen coating as previously described [36]. Briefly, 12 × 10^3^ MDA-MB-231/Hs578T derived cells or 24 × 10^3^ 4T1 derived cells were incubated in 200 μL of medium for 45 or 90 min at 37°C, respectively. Then, cells were washed with PBS, fixed with 100 μL of methanol, stained with 100 μL of 0.5% crystal violet and washed with PBS. Crystal violet was resuspended in 60 μL of 10% methanol and 5% glacial acetic acid solution. Absorbance at 620 nm was determined using an ELISA Multiskan FC (Thermo Scientific). Data were normalized to protein levels from lysates prepared using 1.5 × 10^5^ cells from the plates used for each assay. Collagen coated plates were made by incubation with 200 μL of collagen for 1 hour at room temperature.

### Wound healing assay

For wound healing assay, 3 × 10^5^ MDA-MB-231 shRNA Scramble or shRNA CTBP1 cells were seeded in 12-wells tissue culture plates. After 36 hours, when cells reached confluence, a straight wound line was drawn using a tip and floating cells were removed with PBS washes. For 4T1 cells, 5 × 10^4^ cells were seeded in 12-wells tissue culture plates. After 24 hours, cells were transfected as previously described [10] using 2 μg of pcDNA3 or pcDNA3 CTBP1 vectors. Twenty-four hours later, a wound was made as described above. In both cases, cells were maintained in 1 mL of medium at 37°C 5% CO_2_ for 16 hours in the case of MDA-MB-231 cells, or for 9 hours in the case of 4T1 cells. Pictures were obtained using Q-Color5 Digital Camera (OLYMPUS) at the indicated times after scrape and wound closure quantification was performed using ImageJ 1.48.

### MeS murine model and orthotopic xenograft

Forty-four female *nu*/*nu* mice (4 weeks old) were fed *ad libitum* for 16 weeks with CD (3120 kcal/kg, 5% fat, *n* = 22) or HFD (4520 kcal/kg, 37% fat, *n* = 22) generated supplementing chow food with 32% of bovine fat first juice (Fatty, Buenos Aires, Argentina) [10]. Body weight was determined once a week. After 10 weeks mice were divided randomly in two subgroups and inoculated with 4.8 × 10^6^ MDA-MB-231 shRNA Scramble or MDA-MB-231 shRNA CTBP1 cells in the MFP. Tumor size was measured 3 times a week and its volume was calculated as previously described [10]. Six weeks after inoculation mice were sacrificed and tumor, lung, liver and blood samples were collected. IHC and histological analysis were performed in 5 μm tissue sections using H&E. All animal experiments were housed under pathogen free conditions following the IBYME’s animal care guidelines.

### Tumor and lung tissue samples processing for RT-qPCR

Tumor and lung samples were homogenized in Tri Reagent (Genbiotech, Buenos Aires, Argentina) using Dremel MultiPro 395 and RNA isolation was performed as previously described [10].

### Clonogenic assay and circulating tumor cells (CTCs) detection

After 42–45 days post-injection, mice blood samples were collected by direct heart puncture and anti-coagulated using 60 μL 0.5 M EDTA pH = 8. Blood cells were harvested by centrifugation and red blood cells were lysed by 4 rounds of incubation with ammonium chloride potassium (ACK) lysis buffer. Then, blood cells were cultivated in 3 wells of a 12-well tissue culture plate with 750 μL of DMEM supplemented with 10% FBS, 1 µg/mL puromycin and antibiotics for eleven days. Pictures were obtained using Q-Color5 Digital Camera (OLYMPUS).

### Statistical analysis

All results are given as mean and standard deviation (SD) of at least three independent experiments unless stated otherwise. Student *t* tests were used to ascertain statistical significance with a threshold of *p* < 0.05. For *in vivo* experiments, two-way ANOVA followed by Tuckey test were performed. Shapiro–Wilk and Levene tests were used to assess normality and homogeneity of variances. ^*^p < 0.05; ^**^p < 0.01;^***^p < 0.001.

## SUPPLEMENTARY MATERIALS TABLES





## Abbreviations

ACK: ammonium chloride potassium
BrCa: breast cancer
CD: control diet
CTC: circulating tumor cell
EMT: epithelial-to- mesenchimal transition
FBS: fetal bovine serum
HDL-C: high density lipoprotein cholesterol
HFD: high fat diet
H&E: hematoxylin and eosin
MFP: mammary fat pad
miRNA: microRNA
MeS: metabolic syndrome
NAD^+^: oxidized nicotinamid adenin dinucleotid
NADH: reduced nicotinamid adenin dinucleotid
RT-qPCR: reverse transcription polymerase chain reaction
TNBC: triple negative breast cancer
WB: western blot
