# Supplementary material for: CTBP1 and metabolic syndrome induce an mRNA and miRNA expression profile critical for breast cancer progression and metastasis
Source: Oncotarget. 2018 Feb 13;9(17):13848–58. doi: 10.18632/oncotarget.24486 (PMC5862620; doi:10.18632/oncotarget.24486)
Supplement: Supplementary file 1 [file oncotarget-09-13848-s001.pdf]

CTBP1 and metabolic syndrome induce an mRNA and miRNA expression profile critical for breast cancer progression and metastasis

SUPPLEMENTARY MATERIALS

Supplementary Table 1: Gene ontology analysis of miRNAs regulated by CTBP1 in MeS mice. See Supplementary_Table_1

Supplementary Table 2: Primer sequences used in this work

Primer	Sequence	$T_{annealing}$ (°C)
RT-β Actin Fw	AAGATCATTGCTCCTCCTGAGC	60
RT-β Actin Rv	CATACTCCTGCTTGCTGATCCA	60
RT-Col17A1 Fw	CACTCCAGGACCCAAAGG	60
RT-Col17A1 Rv	TGACAGCAATACTTCTTCTCC	60
RT-CTBP1 Fw	TACAGCGAGCAGGCATCC	60
RT-CTBP1 Rv	TGGTCCTTGTTGACACAGTTC	60
RT-CTBP2 Fw	CCTTAGCACAAGCCCTCAAG	60
RT-CTBP2 Rv	TGCTCACTGTACCAGGCAGT	60
RT-GAPDH Fw	CAGTCAGCCGCATCTTCTTTTG	60
RT-GAPDH Rv	ACCAGAGTTAAAAGCAGCCCT	60
RT-FABP4 Fw	GGAAGTGACGCCTTTCATGA	60
RT-FABP4 Rv	GGATGGAAAATCAACCACCA	60
RT-ITGA1 Fw	GTAATCTCACTTCTTCTGACATC	58
RT-ITGA1 Rv	CAGCATTAACAGCAACAATCC	58
RT-ITGB4 Fw	CGCCTGACTGCTGGTGTG	60
RT-ITGB4 Rv	GGGATGTTGAGCCGATGC	60
RT-ITGB6 Fw	CCTGGCTATTCTTCTCATC	58
RT-ITGB6 Rv	CTGTAGAGTGGATTGGTTC	58
RT-miRNA Rv Stemloop	TGGTGCAGGGTCCGAGGTATT	
RT-let7-7e-3p STEM	GTCTCCTCTGGTGCAGGGTCCGAGGTATTCGCACCAGAGGAGACGGAAAG	
RT-let7-7e-3p Fw	CGCCCACTATACGGCCTCCTAG	67
RT-miR 17-5p STEM	GTCTCCTCTGGTGCAGGGTCCGAGGTATTCGCACCAGAGGAGACCTACCT	
RT-miR 17-5p Fw	GGATGGCAAAGTGCTTACAGTGC	70
RT-miR 103a-3p STEM	GTCTCCTCTGGTGCAGGGTCCGAGGTATTCGCACCAGAGGAGACTCATAG	
RT-miR 103a-3p Fw	GGGGAGCAGCATTGTACAGGG	67
RT-miR 146a-5p STEM	GTCTCCTCTGGTGCAGGGTCCGAGGTATTCGCACCAGAGGAGACAACCCA	
RT-miR 146a-5p Fw	GGGCGGGTGAGAACTGAATTCC	70
RT-miR 191-5p STEM	GTCTCCTCTGGTGCAGGGTCCGAGGTATTCGCACCAGAGGAGACCAGCTG	
RT-miR 191-5p Fw	GCGGCAACGGAATCCCAAAAG	70
RT-miR 194-1-5p STEM	GTCTCCTCTGGTGCAGGGTCCGAGGTATTCGCACCAGAGGAGACTCCACA	
RT-miR 194-1-5p Fw	CCGGCGGTGTAACAGCAACTC	70
RT-miR 223-3p STEM	GTCTCCTCTGGTGCAGGGTCCGAGGTATTCGCACCAGAGGAGACTGGGGT	
RT-miR 223-3p Fw	GGGCGGCTGTCAGTTTGTC	70
RT-miR 378a-3p STEM	GTCTCCTCTGGTGCAGGGTCCGAGGTATTCGCACCAGAGGAGACGCCTTC	
RT-miR 378a-3p Fw	GGCGGACTGGACTTGGAGTC	67
RT-miR 381-5p STEM	GTCTCCTCTGGTGCAGGGTCCGAGGTATTCGCACCAGAGGAGACATATAC	
RT-miR 381-5p Fw	GCGGAGCGAGGTTGCCC	72
RT-miR 494-3p STEM	GTCTCCTCTGGTGCAGGGTCCGAGGTATTCGCACCAGAGGAGACGAGGTT	
RT-miR 494-3p Fw	GGGCGGTGAAACATACACGGG	67
RT-miR 522-5p STEM	GTCTCCTCTGGTGCAGGGTCCGAGGTATTCGCACCAGAGGAGACCAGAAAG	
RT-miR 522-5p Fw	GGGACGGGCTCTAGAGGGAAG	72
RT-PRSS2 Fw	TGGGTGGTGTCAGCAGGTC	60
RT-PRSS2 Rv	ACGGCAGGTGTGGAGAGC	60
RT-TGM2 Fw	GACCAGCACCCCATTGAC	58
RT-TGM2 Rv	CCAGGGCGATGTACTTGTC	58
