# Supplementary material for: CTBP1 and metabolic syndrome induce an mRNA and miRNA expression profile critical for breast cancer progression and metastasis
Source: Oncotarget. 2018 Feb 13;9(17):13848–58. doi: 10.18632/oncotarget.24486 (PMC5862620; doi:10.18632/oncotarget.24486)
Supplement: Supplementary file 2 [file oncotarget-09-13848-s002.docx]

Supplementary Table 1: Gene ontology analysis of miRNAs regulated by CTBP1 in MeS mice.

|  | **CTBP1-regulated miRNA** | | | | | | | | |  |
| --- | --- | --- | --- | --- | --- | --- | --- | --- | --- | --- |
| **Proliferation-related processes** | **miR-378a-3p** | **miR-146a-5p** | **miR-381-3p** | **miR-223-3p** | **miR-494-3p** | **miR-940** | **miR-433-3p** | **miR-522-3p** | **miR-637** | **miR-1285-3p** |
| Cell cyclemitotic | X | X | X | X | X |  | X | X | X |  |
| DNA replication | X | X | X | X | X |  | X | X | X |  |
| Mitotic M-M G1 phases | X | X | X | X |  |  | X | X | X |  |
| Mitotic G1-G1 S phases | X | X | X | X | X |  | X | X |  |  |
| Mitotic prometaphase | X | X | X | X |  |  | X | X | X |  |
| M phase | X | X | X | X |  |  | X | X | X |  |
| G1 S Transition | X | X | X | X | X |  | X | X |  |  |
| Mitotic G2-G2 M phases | X | X | X | X | X |  | X | X |  |  |
| G2 M transition | X | X | X | X | X |  | X | X |  |  |
| Regulation of DNA replication | X | X | X | X | X |  | X | X |  |  |
| S phase | X | X | X |  | X |  | X | X |  |  |
| Cyclin E associated events during G1 S transition | X | X | X |  | X |  | X | X |  |  |
| Cyclin A CDK2-associated events at S phase entry | X | X | X |  | X |  | X | X |  |  |
| Regulation of APC C activators between G1 S and early anaphase | X | X | X | X |  |  | X | X |  |  |
| APC C-mediated degradation of cell cycle proteins | X | X | X | X |  |  | X | X |  |  |
| Regulation of mitotic cell cycle | X | X | X | X |  |  | X | X |  |  |
| Cell cycle checkpoints | X | X | X | X |  |  | X |  |  | X |
| DNA replication pre-iniciation | X | X | X | X |  |  | X | X |  |  |
| M G1 transition | X | X | X | X |  |  | X | X |  |  |
| G1 phase |  | X |  | X | X |  | X | X |  |  |
| Cyclin D associated events in G1 |  | X |  | X | X |  | X | X |  |  |
| G0 and early G1 | X | X | X | X |  |  |  | X |  |  |
| Synthesis of DNA | X | X | X |  | X |  |  |  |  |  |
| Stabilization of p53 | X | X | X |  |  |  |  |  |  | X |
| p53-dependent G1 DNA damage response | X | X | X |  |  |  |  |  |  |  |
| p53-dependent G1 S DNA damage checkpoint | X | X | X |  |  |  |  |  |  |  |
| G1 S DNA damage checkpoints | X | X | X |  |  |  |  |  |  | X |
| p53-independent G1 S DNA damage checkpoint | X | X | X |  |  |  |  |  |  |  |
| E2F mediated regulation of DNA replication |  |  |  | X | X |  |  |  |  |  |
| G2 M checkpoints |  | X | X |  |  |  |  |  |  |  |
| **Tumor progression-related processes** |  | | | | | | | | |  |
| Degradation of Beta-catenin by the destruction complex | X | X | X | X | X |  | X | X |  |  |
| Adherent junctions interactions |  | X | X | X | X | X |  | X |  |  |
| Axon guidance | X | X | X | X | X | X | X | X | X |  |
| Cell surface interactions at the vascular wall | X | X | X | X | X | X | X | X | X |  |
| Cell-cell communication | X | X | X | X | X | X | X | X |  |  |
| Cell junction organization | X | X | X | X | X | X |  | X |  |  |
| Integrin cell surface interactions |  | X | X | X | X | X | X | X |  |  |
| Cell-cell junction organization | X | X | X | X | X | X |  | X |  |  |
| Signaling by Wnt | X | X | X | X | X |  | X | X |  |  |
| Gap junction trafficking and regulation |  | X |  |  | X | X | X |  |  |  |
| Tigh junction interactions | X | X | X |  | X |  |  |  |  |  |

GeneChip miRNA 4.0 Affymetrix was hybridized to total RNA isolated from CTBP1 depleted or control xenograft tumors generated in mice fed with HFD. Reactome analysis using miRSystem tool was performed to identify CTBP1-regulated miRNAs involved in process associated to tumor growth and progression.
